# Ferric Citrate Hydrate as a Phosphate Binder and Risk of Aluminum Toxicity

**DOI:** 10.3390/ph7100990

**Published:** 2014-09-26

**Authors:** Ajay Gupta

**Affiliations:** 1 Division of Nephrology and Hypertension, University of California Irvine Medical Center, Orange, CA 92868-3217, USA; E-Mail: ajayg1@uci.edu; Tel.: +1-562-809-8899; Fax: +1-702-974-1001; 2 Rockwell Medical Inc., 30142 S. Wixom Road, Wixom, MI 48393, USA

**Keywords:** Ferric citrate, aluminum toxicity, CKD

## Abstract

Ferric citrate hydrate was recently approved in Japan as an oral phosphate binder to be taken with food for the control of hyperphosphatemia in patients with chronic kidney disease (CKD). The daily therapeutic dose is about 3 to 6 g, which comprises about 2 to 4 g of citrate. Oral citrate solubilizes aluminum that is present in food and drinking water, and opens the tight junctions in the intestinal epithelium, thereby increasing aluminum absorption and urinary excretion. In healthy animals drinking tap water, oral citrate administration increased aluminum absorption and, over a 4-week period, increased aluminum deposition in brain and bone by about 2- and 20-fold, respectively. Renal excretion of aluminum is impaired in patients with chronic kidney disease, thereby increasing the risk of toxicity. Based on human and animal studies it can be surmised that patients with CKD who are treated with ferric citrate hydrate to control hyperphosphatemia are likely to experience enhanced absorption of aluminum from food and drinking water, thereby increasing the risk of aluminum overload and toxicity.

## 1. Introduction

Ferric citrate hydrate (Riona^®^, Japan Tobacco Inc, Tokyo, Japan) was recently approved by the Japanese Ministry of Health, Labour and Welfare, as a phosphate binder for the improvement of hyperphosphatemia both in patients with dialysis and in patients with non‑dialysis dependent chronic kidney disease (CKD) [1,2]. The doses of elemental iron III [Fe(III)] as ferric citrate needed for effective phosphate binding are 2- to 30-fold greater than the therapeutic doses of Fe(II) salts needed for iron repletion [1]. The usual initial dose is 500 mg of ferric citrate hydrate 3 times a day immediately after meals. Thereafter, the dosage should be adjusted based on the degree of symptoms and on the serum phosphorus concentration. The maximum daily dose is 6000 mg. Ferric citrate hydrate comprises approximately 16% to 18% iron, 19% water, and 63% to 65% citrate by weight. In order to administer sufficient Fe(III) for phosphate binding, large doses of citrate are also administered, with citrate serving as a ligand for Fe(III). The maximum daily oral dose of 6000 mg of ferric citrate hydrate would administer about 3900 mg of citrate and 1000 mg Fe(III). Citrate solubilizes and promotes absorption of trivalent metals, such as aluminum, from dietary sources. Renal elimination of aluminum is impaired in patients who have reduced glomerular filtration rate [3].

The aim of this commentary is to demonstrate that large doses of citrate, administered as ferric citrate hydrate 2 to 3 times a day to patients with CKD, are expected to significantly increase aluminum absorption and increase the risk of aluminum toxicity. Regular clinical and laboratory monitoring for aluminum overload and toxicity is needed when ferric citrate hydrate is used as a phosphate binder, especially in patients with a high dietary intake of aluminum.

## 2. Dietary Intake and Metabolism of Aluminum

The contribution of water and food to the daily oral exposure of aluminum and its health consequences have been reviewed by the World Health Organization (WHO) on numerous occasions [4]. Aluminum is the most abundant metallic element and constitutes about 8% of the earth’s crust. Acid environments caused by acid mine drainage or acid rain can cause an increase in the dissolved aluminum content of the surrounding water. The concentrations of aluminum are usually low in ground water but are often high in surface waters, and aluminum levels in drinking water vary according to the levels that are found in the source water and whether aluminum coagulants are used during water treatment. The WHO has not proposed a health-based guideline for aluminum in drinking water because of the limitations of the animal data. However, “practicable levels” of ≤0.1 and ≤0.2 mg/L have been derived for large and small facilities, respectively, based on optimization of the coagulation process in water treatment plants that use aluminum-based coagulants [4].

Aluminum is found in the tissues of all plants and animals, and consequently is naturally present in foods. The concentration of aluminum in foods varies widely, depending upon the product, the type of processing, and the geographical origin [5]. Grain products, processed cheese, tea, herbs, spices, potatoes, spinach and salt-containing aluminum additives have been identified as the major sources of aluminum in daily diets [5]. Herbs and tea leaves are the richest natural dietary sources of aluminum. Tea infusate contains up to 0.5 mg of aluminum per 100 g of infusate. It has been estimated that ingestion of 240 mL of tea with each meal would add 1 to 3 mg of aluminum to the diet [6] and that consumption of 5 cups of tea per day would result in an exposure of 5 to 7 mg of aluminum [7]. Consumption of 1.2 L of tea per day has been shown to markedly increase urinary excretion of aluminum [8]. Additional dietary burden may ensue from use of aluminum-containing food additives. Processed dairy products and flour may be high in aluminum if they contain aluminum-based food additives. The use of aluminum cookware, utensils, and wrappings also can increase the amount of aluminum in food.

The average daily adult aluminum intake in food and water totals approximately 5 milligrams, with a wide range of 1 to 20 mg [9]. Ingestion of certain foods or nonprescription drugs can readily increase the daily aluminum intake by several hundred milligrams [10]. People who ingest aluminum-containing antacids and buffered analgesics may have intake as much as 5 g/day of aluminum [9].

Despite daily dietary exposure to aluminum in food and water, intestinal absorption of aluminum is extremely limited in part due to the poor solubility of aluminum at the pH of the small intestine where aluminum precipitates with phosphates and is unavailable for absorption [11]. The variability in aluminum absorption results from the chemical properties of the element itself, as well as from the formation of various chemical species, which is dependent upon the pH, ionic strength, the presence of competing elements (e.g., silicon), and the presence of chelating agents within the gastrointestinal tract (e.g., citrate).

Normal plasma aluminum concentrations are about 1 to 2 µg/L, with slightly more than 90% bound to transferrin (Tf). The normal tissue distribution of aluminum is approximately 60% in bone, 25% in lung (from inhalation of air), 10% in muscle, 3% in liver, and 1% in brain. More than 95% of aluminum is eliminated by the kidney, with only approximately 2% eliminated in bile [12]. Therefore, patients with CKD are at greatest risk of aluminum toxicity.

Gels containing aluminum were commonly used as phosphate binders between 1970 and 1990. Accumulation of aluminum due to long-term use of these binders in patients with CKD caused serious complications, including encephalopathy, osteomalacia, proximal myopathy, and microcytic anemia [13]. Notably, citrate-enhanced absorption of aluminum in children and young adults was a major concern in the 1980s [14].

## 3. Citrate Promotes Absorption of Dietary Aluminum

**Figure 1:**
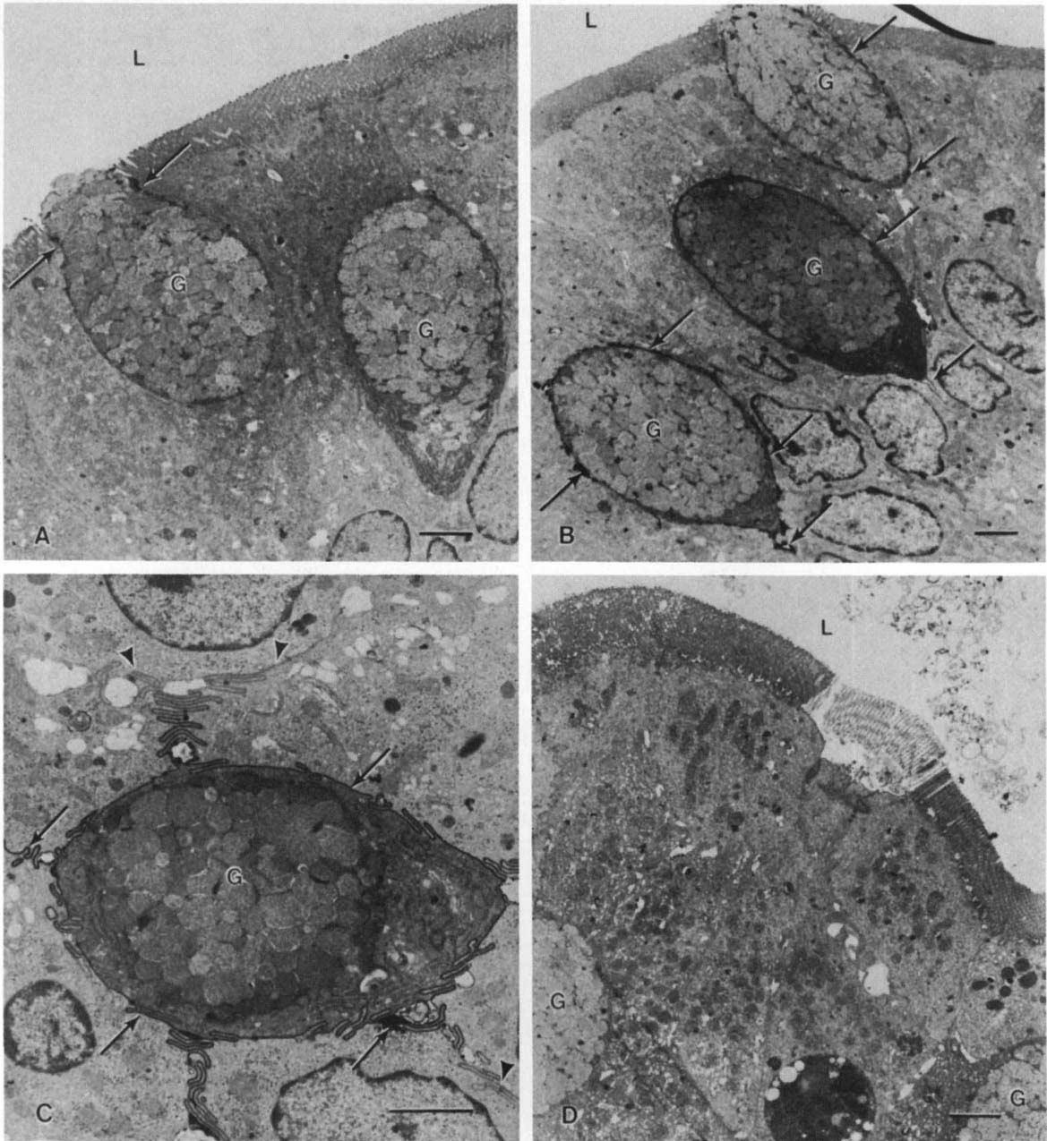
Electron micrograph of isolated duodenal loops. **A**. Following exposure to normal saline, rare ruthenium red deposits could be seen around goblet cells (arrows). No deposits were visible between adjacent columnar epithelial cells (magnification of ×4600), **B**. Following aluminum citrate exposure, dense infiltration of ruthenium red deposits could be visualized around goblet cells (arrows). The mucosal epithelium following both normal saline and aluminum citrate was intact (magnification ×3700). **C**. A goblet cell, at higher magnification, following aluminum citrate treatment. Note the intense, ribbon-like outlining of the entire goblet cell’s intercellular space by ruthenium red which fades away at the junction of the columnar epithelial intercellular space (arrows) (magnification of ×7600). **D**. Aluminum chloride pre-incubation resulted in minimal or no ruthenium red in intercellular spaces but caused some patchy sloughing of mucosa (magnification of ×4400; G, goblet cell; L, lumen; bar represents 2 microns). Reprinted with permission from the International Society of Nephrology [16].

**Figure 2:**
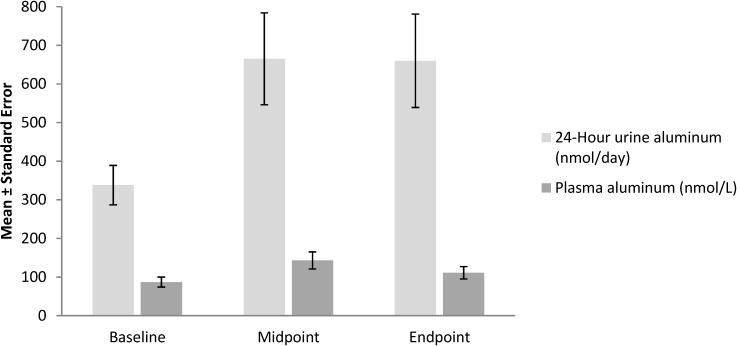
Aluminum absorption during calcium citrate treatment in 26 subjects. Midpoint equals 19 ± 6 days; endpoint equals 38 ± 14 days. Data were derived from Nolan *et al.* [3].

Citrate tri-anion reacts with aluminum to form a complex that is soluble over the wider pH range [15]. Furthermore, citrate chelates extracellular calcium causing disruption of the integrity of intestinal epithelial cell tight junctions (Figure 1), creating a paracellular pathway for the absorption of soluble aluminum citrate complexes [16]. Animal models suggest that citrate‑containing compounds augment absorption of aluminum from tap water and food, causing aluminum accumulation in bone and brain despite normal renal function [17]. Nolan and colleagues [3] measured aluminum levels in plasma and in 24-hour urine collections in 30 healthy women before and during treatment with calcium citrate (800 mg calcium and approximately 2500 mg citrate), administered daily in 2 divided doses (Figure 2). After approximately 2 weeks, plasma levels of aluminum increased from 87 ± 13 to 143 ± 22 nmol/L (*p* = 0.03), and the 24‑hour urinary aluminum excretion increased from 338 ± 51 to 655 ± 119 nmol/day (*p* = 0.002). This highly significant increase in urine and serum aluminum occurred despite a relatively low aluminum content of the municipal water (3.4 ± 1.2 µg/L). Notably, citrate administration had no effect on lead absorption. Alfrey and coworkers [18] concluded that citrate significantly increases absorption of aluminum from dietary sources. Animal models suggest that citrate-containing compounds augment absorption of aluminum from tap water and food, causing aluminum accumulation in bone and brain despite normal renal function [17].

## 4. Citrate Leads to Accumulation of Aluminum in Brain and Bones

There is evidence that citrate-induced absorption of dietary aluminum is associated with accumulation of aluminum in tissues even in the presence of normal renal function. Aluminum concentration was about 2 times higher in brain cortex and 20 times higher in bone, in rats that were administered citric acid in drinking water for 4 weeks [17]. When citric acid was withheld over the subsequent 5 weeks, no decline of aluminum in tissue levels was observed, demonstrating persistent accumulation. Neither serum nor urine levels of aluminum were reported in this study.

Factually, serum aluminum levels alone are not a reliable indicator of the level of its deposition in tissues such as bone, *etc.* Increased aluminum deposition in bone may occur despite low serum aluminum levels [19,20,21]. In a 1996 review, D’Haese *et al.* expounded on the low reliability of the serum aluminum assays [22]. In patients with iron overload (as it occurs with ferric citrate hydrate), serum aluminum level <30 µg/L was accompanied by serious bone accumulation of aluminum [22]. Thus low plasma aluminum levels do not exclude the possibility of aluminum overload and aluminum bone disease. In the wake of this fact, it is imperative that in CKD patients, serum and tissue levels (such as bone, *etc.*) of aluminum be reported in conjunction, to ascertain aluminum toxicity status [23]. A positive desferrioxamine (DFO) test is used as a non-invasive test to diagnose aluminum bone disease [24]. Poignantly, the Yokoyama studies [1,2] did not report any of these parameters.

## 5. Use of Ferric Citrate Hydrate as a Phosphate Binder in Patients with CKD: Source of Citrate and a Risk Factor for Aluminum Toxicity

The amount of citrate that is administered when ferric citrate hydrate is used as a phosphate binder is comparable to, but often far exceeds, the amount that was administered as calcium citrate in the study that was conducted by Nolan and colleagues [3]. Therefore, administration of ferric citrate hydrate would be expected to increase the absorption of dietary aluminum, leading to retention and tissue accumulation of aluminum in patients with CKD, since aluminum cannot be adequately excreted via the renal route in patients with CKD. The amount of aluminum absorbed could be further increased if dietary intake of aluminum is high or if the patient ingests aluminum-containing antacids, buffered analgesics or, aluminum containing phosphate binders.

In a randomized, double-blind, placebo-controlled study of ferric citrate hydrate (Riona) for the treatment of hyperphosphatemia in patients with nondialysis-dependent CKD, 57 Japanese patients received ferric citrate hydrate for up to 12 weeks [1]. Serum phosphate levels decreased from 5.66 ± 0.75 to 4.37 ± 1.3 mg/dL (*p* < 0.001), whereas transferrin saturation (TSAT) increased from 27.2% ± 11.3% to 44.2% ± 20.9% (*p* < 0.001) and serum ferritin levels increased from 69.0 ± 50.9 to 204.0 ± 106.5 ng/mL (*p* < 0.001). The increase in TSAT and ferritin values during treatment with ferric citrate hydrate in apparently iron-replete subjects with CKD over a relatively short 12-week period is remarkable, considering that the oral bioavailability of iron from ferric citrate in iron replete healthy subjects is only about 1.6% [25]. Similar increments in serum TSAT and ferritin were reported in Japanese patients on maintenance hemodialysis who were administered ferric citrate over a 12-week period [2]. This suggests that iron absorption from ferric citrate when administered in large doses is relatively poorly regulated and may circumvent the hepcidin-mediated block in iron absorption. This is consistent with demonstration by Froment and coworkers [16] that citrate chelates extracellular calcium, causing disruption of the integrity of intestinal epithelial cell tight junctions and creating a paracellular pathway for the absorption of soluble aluminum citrate complexes (Figure 1). Opening of the paracellular pathway would be expected to increase both iron and aluminum absorption. Notably, either dietary aluminum intake or urinary, serum and bone tissue aluminum levels were not reported in the studies recently published studies by Yokoyama *et al.* [1,2].

In the first clinical study of ferric citrate hydrate as a phosphate binder, 45 Taiwanese hemodialysis patients were treated with 1 g of ferric citrate 3 times a day with meals [26]. During the short 4-week exposure to ferric citrate, serum ferritin levels increased significantly from 221 ± 115 to 248 ± 143 ng/mL (*p* < 0.03), whereas serum aluminum levels showed a nonsignificant increase from 11.0 ± 6.9 to 12.9 ± 7.5 µg/L. The investigators concluded that “although there is no evidence that ferric citrate causes aluminum toxicity, this issue will require further monitoring, particularly in areas with a high aluminum concentration in drinking water”.

## 6. Interaction between Aluminum and Iron

Aluminum and iron are carried in the plasma by the same carrier protein Tf. Slightly more than 90% of plasma aluminum is associated with Tf, about 7% to 8% is associated with citrate, and <1% is associated with phosphate and hydroxide [12]. Iron and aluminum, when bound to transferrin, enter the cells via transferrin receptors (TfR). Similar values of affinity constant for the binding of TfR to Tf carrying either aluminum or iron have been reported [27]. However, it is the non-Tf bound, low-molecular fraction that is able to cross the blood brain barrier and that is taken up by brain tissue. Therefore, iron loading of Tf would be expected to increase the non-Tf bound fraction of plasma aluminum, further increasing aluminum toxicity.

There is evidence that aluminum toxicity may be mediated in part by iron toxicity. In aluminum-treated rats, the increases in tissue aluminum content were paralleled by elevations of tissue iron in the kidney, liver heart, and spleen, as well as in various brain regions (e.g., frontal lobe, temporal lobe, and parietal cortex and hippocampus) [28]. Additionally, in aluminum‑treated drosophila flies, there was accumulation of large amount of iron and reactive oxygen species (ROS) with elevated superoxide dismutase activity; genetic and pharmacological efforts to reduce ROS or chelate excess iron significantly mitigated aluminum toxicity [29]. This indicates that aluminum toxicity is mediated through production of ROS and iron accumulation. Ferric citrate administration as a phosphate binder would lead to aluminum and iron loading concurrently. The evidence suggests that the iron loading could potentially further aggravate aluminum toxicity.

There is growing evidence of a relationship between aluminum exposure and neurodegenerative diseases, including dialysis encephalopathy, amyotrophic lateral sclerosis, and Alzheimer’s disease (reviewed by Kawahara and Kato-Negishi) [30]. Oligomerization of β-amyloid protein leading to neurotoxicity is the likely key mechanism in the pathogenesis of Alzheimer’s disease, and aluminum may play crucial roles as a cross-linker in β-amyloid oligomerization. Aluminum is sequestered in bone for long periods; therefore, the toxic effects are cumulative [23]. Aluminum can lead to disruption of mineralization and inhibition of bone-cell growth and activity. The clinical manifestations are adynamic bone disease, low bone turnover osteoporosis, osteomalacia, and multiple nonhealing fractures. As blood aluminum level is a relatively poor indicator of tissue accumulation, diagnosis of aluminum accumulation in bone may require a bone biopsy.

## 7. Conclusions

Ferric citrate hydrate is a novel phosphate binder that has recently been approved in Japan for control of hyperphosphatemia in patients with CKD. Doses sufficient to bind phosphate deliver 2 to 4 grams of citrate, and such doses are expected to enhance the absorption of aluminum in diet by solubilizing aluminum and opening the intestinal paracellular pathway for aluminum absorption. Tea and food additives are major contributors to dietary aluminum intake, and both items constitute a significant component of Japanese diet, further increasing the risk of aluminum overload and chronic toxicity. Therefore, patients who are prescribed ferric citrate hydrate should receive dietary counseling to limit dietary aluminum intake and should undergo regular clinical monitoring for early detection of aluminum overload and toxicity.
